# Exploring Tree-Habitat Associations in a Chinese Subtropical Forest Plot Using a Molecular Phylogeny Generated from DNA Barcode Loci

**DOI:** 10.1371/journal.pone.0021273

**Published:** 2011-06-20

**Authors:** Nancai Pei, Ju-Yu Lian, David L. Erickson, Nathan G. Swenson, W. John Kress, Wan-Hui Ye, Xue-Jun Ge

**Affiliations:** 1 Key Laboratory of Plant Resource Conservation and Sustainable Utilization, South China Botanical Garden, Chinese Academy of Sciences, Guangzhou, People's Republic of China; 2 The Graduate University of the Chinese Academy of Sciences, Beijing, People's Republic of China; 3 Department of Botany, National Museum of Natural History Smithsonian Institution, Washington, D.C., United States of America; 4 Department of Plant Biology, Michigan State University, East Lansing, Michigan, United States of America; Montreal Botanical Garden, Canada

## Abstract

Elucidating the ecological mechanisms underlying community assembly in subtropical forests remains a central challenge for ecologists. The assembly of species into communities can be due to interspecific differences in habitat associations, and there is increasing evidence that these associations may have an underlying phylogenetic structure in contemporary terrestrial communities. In other words, by examining the degree to which closely related species prefer similar habitats and the degree to which they co-occur, ecologists are able to infer the mechanisms underlying community assembly. Here we implement this approach in a diverse subtropical tree community in China using a long-term forest dynamics plot and a molecular phylogeny generated from three DNA barcode loci. We find that there is phylogenetic signal in plant-habitat associations (i.e. closely related species tend to prefer similar habitats) and that patterns of co-occurrence within habitats are typically non-random with respect to phylogeny. In particular, we found phylogenetic clustering in valley and low-slope habitats in this forest, indicating a filtering of lineages plays a dominant role in structuring communities in these habitats and we found evidence of phylogenetic overdispersion in high-slope, ridge-top and high-gully habitats, indicating that distantly related species tended to co-occur in these high elevation habitats and that lineage filtering is less important in structuring these communities. Thus we infer that non-neutral niche-based processes acting upon evolutionarily conserved habitat preferences explain the assembly of local scale communities in the forest studied.

## Introduction

Determining the ecological and evolutionary processes underlying community assembly remains a central goal in community ecology. Perhaps nowhere has the debate regarding the assembly of communities been more vigorous than in tropical tree community ecology. Proposed assembly mechanisms invoke the relative importance of niche- [1], [2], [3] and neutral-based [4], [5], [6] processes. Tests of these niche- and neutral-based mechanisms have occasionally focused on the degree to which species are associated with the underlying environment where strong associations are indicative of niche-based mechanisms dominating the assembly process [7], [8]. Recent work has demonstrated that both species and entire clades have strong associations with soil habitats [9]. In other words there may often be substantial phylogenetic signal in plant-soil habitat associations where closely related species tend to be found on similar soils. This suggests that the evolutionary history of species may help explain their present day distribution and co-occurrence patterns along habitat gradients and that niche-based process can be detected using phylogenetic information.

The use of phylogenetic information in plant community ecology has dramatically increased since the pioneering work of Webb [10]. A conceptual framework has emerged from this literature that is designed to identify the relative influence of niche-based versus neutral processes during the assembly of communities. Specifically this conceptual framework integrates the phylogenetic signal in species traits or niches (i.e. the degree of trait or niche similarity between closely related species) with patterns of community phylogenetic structure (i.e. phylogenetic clustering or overdispersion) in order to infer the relative influence of habitat filtering, limiting similarity or neutrality during community assembly (Table 1).

**Table 1:** A conceptual framework integrating the degree of phylogenetic signal in plant-soil habitat associations and the phylogenetic structure of the assemblage.

	Phylogenetically Clustered Assemblage	Phylogenetically Random Assemblage	Phylogenetically Overdispersed Assemblage
**Phylogenetic Signal in Plant-Soil Habitat Associations**	Habitat Filtering	Neutrality	Limiting Similarity
**Phylogenetic ‘Anti-Signal’ in Plant-Soil Habitat Associations**	Limiting Similarity	Neutrality	Habitat Filtering

Niche-based processes (i.e. habitat filtering of limiting similarity) are indicated by a non-random phylogenetic structure of the assemblage, but which processes can only be inferred by considering the degree of phylogenetic signal in plant-soil habitat associations. A Neutral model is supported when the assemblage is random with respect to phylogeny regardless of the degree of phylogenetic signal in plant-soil habitat associations. (Adapted from Kraft NJB, Cornwell WK, Webb CO, Ackerly DD (2007) Trait evolution, community assembly, and the phylogenetic structure of ecological communities. Am Nat 170: 271–283).

In tropical tree community ecology phylogenetic analyses of communities have generally used one of three approaches: (*i*) they have examined only the phylogenetic structure of communities [10], [11], [12], [13]; (*ii*) they have examined only the phylogenetic signal in plant-soil associations [9] or (*iii*) they have integrated patterns of phylogenetic signal in functional traits with patterns of phylogenetic community structure [14], [15]. While many of these studies have inferred the relative influence of niche-based or neutral processes, none has successfully implemented the conceptual framework presented in Table 1 where phylogenetic signal of habitat associations is integrated with patterns of phylogenetic community structure. This is surprising because a popular explanatory niche-based mechanism for the maintenance of tropical forest tree species diversity and community assembly is habitat partitioning [16], [17], [18]. This mechanism is expected to lead to non-random patterns of species-habitat associations. If these associations have strong phylogenetic signal or ‘anti-signal’ (i.e. closely related species have non-randomly diverged in their soil habitat associations), then niche-based processes should result in non-random phylogenetic community structure (Table 1).

Although there has been no study that has explicitly linked levels of phylogenetic signal in tropical tree-habitat associations with patterns of phylogenetic community structure, there have been two studies that have examined the phylogenetic community structure of tropical trees in different habitats. Both of these studies have been conducted in the 50-ha Barro Colorado Island (BCI) forest dynamics plot in Panama. The first study was conducted by Kembel and Hubbell [11] who found that species in ‘dry plateau’ and ‘young’ forest habitats tended to be more phylogenetically related than expected by chance (i.e. phylogenetically clustered) and species in the ‘slope’ and ‘swamp’ habitats tended to be more distantly related than expected by chance (i.e. phylogenetic overdispersion). Kembel and Hubbell [11] inferred that in the former case environmental filtering acting on evolutionarily conserved traits was the community assembly mechanism and biotic interactions acting on evolutionarily conserved traits was the community assembly mechanism in the latter case. This work was important because a previous study from this forest had found little evidence for species-specific habitat associations [8]. Thus the discrepancies between the two studies suggested that analyses that include phylogenetic data may refine our understanding of the role of niche-based processes during tropical tree community assembly.

The study by Kembel and Hubbell [11] utilized a phylogenetic hypothesis estimated by an informatics tool called Phylomatic [19]. This phylogeny contained many unresolved relationships (i.e. soft polytomies) particularly within families. It was unclear at the time of the Kembel and Hubbell [11] study how much this lack of phylogenetic resolution influenced their results and inferences. Recently Kress et al. [13] revisited the analyses of Kembel and Hubbell [11] using a highly resolved molecular phylogeny constructed from three DNA barcode loci. They concluded that many of the results in the original Kembel and Hubbell [11] study were not supported when using the resolved molecular phylogeny. Further the results from Kress et al. [13] showed that the work by Kembel and Hubbell [11], which relied upon the poorly resolved Phylomatic phylogeny, generally underestimated the degree of phylogenetic structuring of the tree communities in the seven BCI habitats. The stronger patterns of structuring found was taken as evidence that terminal phylogenetic resolution provided by a molecular phylogeny generated using DNA barcode loci is critical for identifying the underlying phylogenetic structure of communities and that a lack of resolution may lead to type II statistical errors as previously suggested by Swenson [20]. Thus the implementation of phylogenetic information that allows for species-level resolution should improve the quality of community phylogenetic analyses.

The majority of the phylogenetic analyses of tree communities that have been performed to date are from North and South America or in Southeast Asia. Ideally a greater breadth of forests that have distinctive biogeographic histories should be studied in order to elucidate whether or not any general trends or emergent properties exist. For example, local species richness is known to be highly correlated with regional scale species richness [21], [22]. Thus regional scale differences in species richness likely plays a predominant role in determining differences in local scale richness from region to region. That said, it is still of interest how local scale processes ‘scale-up’ to produce differences in regional species richness and/or whether the strength of niche-based or neutral processes varies between local communities occurring in different regions [23]. In other words, are local scale niche-based processes such as habitat filtering uniformly important in two regions with vastly different levels of biodiversity? In order to answer such a question, researchers must continue to sample, analyze and compare the phylogenetic structure of tree communities from as many regions as possible.

Here we utilize a molecular phylogeny constructed from three DNA barcode loci (*rbcL*, *matK*, and *trnH-psbA*) representing 183 woody plant species in the 20-ha Dinghushan forest dynamics plot in China. The phylogeny, observed spatial distribution patterns for the 188 species in different habitats and a conceptual framework that integrates the degree of phylogenetic signal in plant-habitat associations with the phylogenetic structure of communities are used to ask the central question of whether niche-based (i.e. habitat partitioning or limiting similarity) or neutral processes determine the assembly of species in this subtropical seasonal forest? In answering this central question we also compare and contrast results of the analyses when utilizing a molecular phylogeny versus a phylogeny derived from Phylomatic.

## Materials and Methods

### Research site and DNA sequencing

The present study took place in the Dinghushan forest dynamics plot (DHS FDP) located within the Dinghushan National Nature Reserve (23°09′21″–23°11′30″N, 112°30′39″–112°33′41″E) in Guangdong province, south China. The DHS FDP is a key node in the Chinese Forest Biodiversity Monitoring Network and a part of the Center for Tropical Forest Science (CTFS) global network of forest dynamics plots, The DHS FDP is a subtropical forest with a mean annual rainfall of 1,985 mm. A total number of 71,336 woody stems greater than or equal to 1 cm diameter at breast height have been mapped and identified to species in the 400 m×500 m plot. There are 183 species (188 taxa) of trees and shrubs in the DHS FDP. These 183 species represent 24 orders, 51 families, and 110 genera (38 genera containing ≥ two species; 10 genera containing ≥ four species; and two genera containing ≥ eight species).

A molecular community phylogeny was generated for the 183 species in the DHS FDP by sequencing three DNA barcoding loci (*rbcL*, *trnH-psbA*, and *matK*). DNA sequences were generated for 1–2 tagged individuals located within the plot. Genomic DNA was extracted from leaf and/or bark tissue using a standard CTAB protocol [24]. Standard barcode primers (*rbcL*, *matK*, and *trnH-psbA*) suggested by the Consortium for the Barcode of Life (http://barcoding.si.edu/) were used in the study. The PCR cycling conditions utilized in this study were as follows: *rbcL* and *trnH-psbA* used 95°C for 3 min, (95°C 30 sec, 53°C 45 sec, 72°C 1 min)×34 cycles, 72°C 7 min, while *matK* required lower annealing temperatures, longer extension time and more cycles (95°C 3 min (95°C 30 sec, 51°C 45 sec, 72°C 1.5 min)×38 cycles, 72°C 7 min) [25], adding a final concentration of 5% for DMSO. Sequences of *rbcL* (∼650 bp for the sequence length) which can be sequenced via one reaction, had 1-fold coverage, but the *matK* (∼900 bp) and *trnH-psbA* (280–870 bp) had 2-fold coverage. All DNA sequence data were submitted to GenBank and their accession numbers are provided in Table S1.

### Phylogenetic reconstruction

**Figure 1:**
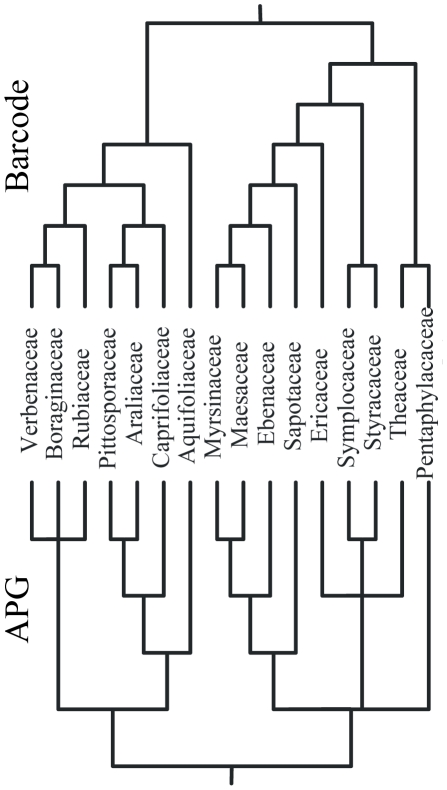
A comparison of the family-level relationships within the Asterid clade. The topology on the left-hand side represents the phylogenetic relationships of families obtained from the APG III consensus phylogeny, while the topology on the right-hand side represents the DHS phylogeny generated with the ML analysis of the barcode sequence data.

We reconstructed two types of community phylogenies representing the 183 plant species found in the DHS FDP. The first phylogeny we generated was a molecular phylogeny using the three sequenced barcode loci described in the previous section. A DNA supermatrix was generated that contained all three markers (*rbcL* + *matK* + *trnH-psbA*) ([13]; see also Text S1 for more detail on alignment and matrix construction). The DNA supermatrix was then analyzed using RA×ML [26] via the CIPRES supercomputer cluster [27] to infer a maximum likelihood (ML) community phylogeny. Node support was estimated using bootstrap values with nodes with less than 50% support being collapsed into soft polytomies. The familial topology in this molecular phylogeny was concordant with that observed in the APG III classification. In Fig. 1 we show a comparison of the family-level relationships within the Asterids clade between our ML analysis of the barcode sequence data and that derived by the APG III.

A second phylogenetic tree was generated for this work using the informatics tool Phylomatic [19]. This tree ‘grafts’ taxonomic relationship to a stored phylogenetic ‘backbone’ is generally resolved to the family level. Thus relationships between species within a genus and genera within a family are generally level unresolved. Community phylogenies derived from Phylomatic are the typical approach in community phylogenetics investigations, because molecular phylogenies of most tropical taxa are not available. As such the Phylomatic tree in this study was generated in order to compare whether any information would be lost if only a Phylomatic tree, exhibiting lower rates of resolution, was utilized.

### Habitat types and spatial scales classification

**Figure 2:**
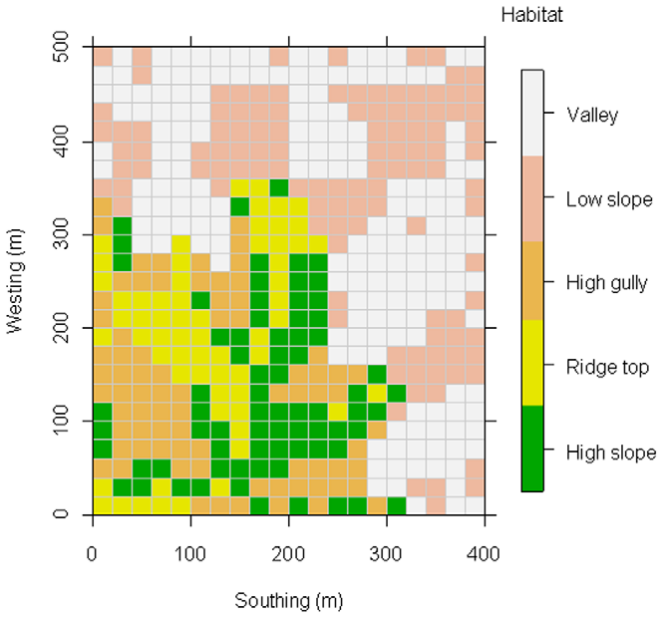
The spatial distribution of the five habitat types in the 20-ha Dinghushan plot. Colors represent different habitat types at the spatial scale of 20 m×20 m.

Five habitat types in the DHS FDP were classified using the topographic variables slope, elevation and convexity [28], [29]. In particular, habitats were classified using a quantitative method where the observed slope and elevation was compared to the plot median value. The specific classification scheme is given in Table 2. The quantitative classification of habitat types allows for them to be ordered by similarity, as valley (V), low-slope (LS), high-gully (HG), ridge-top (RT) and high-slope (HS). A habitat type was assigned to each given 20×20 m quadrat in the DHS FDP. Topographical variations in the DHS FDP are larger than that of the BCI forest plot in Panama (Table 2, and Fig. 2). Thus it is difficult to compare the habitat types of the two plots. The majority of the analyses were conducted by dividing the 20-ha plot into 500 20 m×20 m quadrats. Two additional spatial scales were used, specifically 40 m×40 m and 100 m×100 m. In sum, five habitat types and three spatial scales were used to quantify the community phylogenetic structure in the DHS FDP.

**Table 2:** Criterions of habitat classification, areas of each habitat, total numbers of species and stems ≥1-cm d.b.h. in 2005 census, and total stem densities by habitat for the 20-ha Forest Dynamics Plot of Dinghushan, China.

Habitat	Valley	High-gully	Low-slope	High-slope	Ridge-top
Area (ha)	6.92	3.08	4.60	2.92	2.48
Slope (degrees)	<33	≥33	≥33	≥33	<33
Elevation (m)	<326.3	≥326.3	<326.3	≥326.3	≥326.3
Convexity (degrees)	all	<0	all	>	>0
Mean ± s.e. (species diversity)	25.58±0.46	28.27±0.88	27.57±0.48	34.73±0.84	27.53±0.85
Total number of species	149	133	135	135	105
Total number of stems [density (no.ha^−1^)]	19,501 (2828.06)	11,052 (3588.31)	17,215 (3742.39)	14,174 (4854.11)	9,394 (3787.90)

*Notes*: Valley (slope < median (slope), elevation < median (elevation)); Low-slope (slope ≥ median(slope), elevation < median(elevation)); High-slope (slope ≥ median(slope), elevation ≥ median(elevation), convexity >0); High-gully (slope ≥ median(slope), elevation ≥ median(elevation), c≥onvexity <0); Ridge-top (slope < median(slope), elevation ≥ median(elevation), convexity >0).

Median slope  = 33 degrees; Median elevation  = 326.3 m.

### Habitat association – randomization tests and phylogenetic signal

In order to quantify the degree to which individual species in the DHS FDP are associated with specific habitat types we used the habitat randomization procedure described in Harms et al. [10]. Specifically a torus translation was utilized where the habitat map was ‘rotated’ or iterated. During each iteration, a ‘null’ species-habitat association was calculated for each of the 99 most common tree species. These 99 species were selected because they were common enough (*n*>20) to provide a robust estimate of their habitat association. This generated a null distribution to which we could compare the observed association. Each simulated map included 173 valley, 77 high-gully, 115 low-slope, 73 high-slope, and 62 ridge-top quadrats. This randomization procedure maintains the observed spatial autocorrelation of both the habitat data and the species distributions.

We also quantified the phylogenetic signal in plant-habitat associations in order to implement the conceptual framework presented in Table 1. Phylogenetic signal was measured on the median habitat in which individuals of each species are found using the five habitat categories as ordered variables as described in the above section. The descriptive statistic *K* presented in Blomberg et al. [30] was used to measure the phylogenetic signal in habitat associations. The significance of the observed *K* value was determined using a permutation test. Specifically, the names of taxa were randomized across the tips of the phylogeny 999 times. During each iteration, a null *K* value was calculated and recorded. This generated a distribution of 999 null *K* values to which the observed could be compared.

### Community phylogenetic structure analyses

One of the 19 equally likely phylogenetic trees of the three-locus ML analysis of 183 species was randomly selected to use in the present community phylogenetic analysis. Non-parametric rate smoothing in the R package ‘ape’ [31], [32] was used to generate an ultrametric phylogeny. This ultrametric barcode phylogeny was used for all subsequent community phylogenetic analyses.

Using both the molecular and Phylomatic phylogenies, we quantified the Net Relatedness Index (NRI) and the Nearest Taxon Index (NTI) [33], [34] for each 400 m^2^ quadrats (n = 500). The NRI and NTI are calculated as follows:
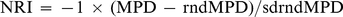


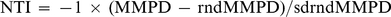
Where MPD represents the mean pairwise phylogenetic distance between all taxa within a local assemblage and MMPD represents the mean phylogenetic distance for each taxa to its nearest relative within a local assemblage. The rndMPD and rndMMPD represent the mean MPD and mean MMPD from 999 randomly generated assemblages. An independent swap null model was used to generate these 999 random assemblages [35]. This is the same null model as the ‘constrained’ null model in Kembel and Hubbell [11]. The NRI is generally considered to be a ‘basal’ metric while the NTI is generally considered to be a ‘terminal’ metric. Negative values of both metrics indicate phylogenetic overdispersion. In other words species in local assemblages are more phylogenetically diverse than expected by chance. Positive values of both metrics indicate phylogenetic clustering. In other words species in local assemblages are more closely related than expected by chance.

Because the NRI and NTI values in the 500 quadrats were spatially autocorrelated, we estimated the mean NRI and NTI values within habitats using simultaneous spatial autoregression analyses. We used generalized least-squares models with a first-order spatial neighbor SAR covariance structures in S+SpatialStats [36] to perform these analyses. Next, following Kembel and Hubbell [11], we defined habitats for each 400 m^2^ quadrat and tested whether each habitat type tended to contain quadrats that were on average phylogenetically clustered, overdispersed or random using t-tests.

Then, following the methods of Kress et al. [13], we asked whether the results for each individual habitat type generated from the molecular phylogeny and the Phylomatic phylogeny were significantly different. For all of the 500 quadrats combined, we compared the NRI and NTI values quantified from the molecular phylogeny to those calculated from the Phylomatic phylogeny using a paired t-test.

Lastly, we performed all analyses using the species lists in the five habitats as individual communities and the forest plot species list as the species pool. This analysis was designed to address whether or not the entire species assemblage in a habitat was phylogenetically non-random.

## Results

### Habitat-species association and community assembly

The habitat association tests recovered 52 significant positive and negative plant-habitat associations out of a potential 495 species-habitat combinations. Thus 10.5% of the tests were positive, which is greater than the expected false discovery rate of 5%. There were 29 significant positive or negative associations in the habitats that were phylogenetically overdispersed (23 for the high-slope habitat and 5 for the high-gully habitat) or phylogenetically clustered (one for the low-slope habitat, but no significant positive or negative associations in the valley habitat). Another 23 significant positive or negative associations were found in the ridge-top habitat which contained phylogenetically random assemblages. A total of 52 of the 99 most common species had a significantly positive or negative association with at least one habitat type (Table 3). The detailed results regarding which species were associated with individual habitat types are provided as Supplemental Material (Text S2).

**Table 3:** Randomized-habitat tests for habitat associations on the 20-ha Forest Dynamics Plot of Dinghushan, China.

Habitat association	99 species	19 species	Habitat association	99 species	19 species
Valley +	0	0	Valley -	0	0
High-gully +	4	1	High-gully -	1	0
Low-slope +	1	1	Low-slope -	0	0
High-slope +	22	2	High-slope -	1	0
Ridge-top +	11	4	Ridge-top -	12	4
Total +	38	8	Total -	14	4

The first column contains results for the 99 common species for which there were ≥20 stems in the plot in the 2005 census. The second column contains results for the 19 most abundant species, all of which had ≥1000 stems in the plot in the 2005 census. For each habitat, “+” indicates significant positive association and “−” indicates significant negative association (two-tailed test, α = 0.05).

The 19 most abundant species accounted for 74.77% of all stems in the DHS FDP. Of these species 12 had at least one positive or negative habitat association, while 7 were not significantly associated with a habitat (Table 3). We detected significant difference (F = 26.414, *P*<0.001) in species richness between the forest communities that were phylogenetically clustered (30.28±0.54, mean ± se) and those that were phylogenetically overdispersed (26.38±0.34) using ANOVA. We further found that the high-slope habitat had the highest species richness (34.73±0.84), followed by habitats of the high-gully (28.27±0.88), the low-slope (27.57±0.48), the ridge-top (27.53±0.85) and the valley (25.58±0.46) (Table 2).

### Phylogenetic signal in habitat associations

We utilized the descriptive statistic *K* to quantify the phylogenetic signal in habitat associations using the median habitat type for all individuals of a species and treating habitat type as an ordered variable. The observed *K* value was 0.80 and this value was compared to a distribution of 999 null *K* values generated with a permutation test. The observed *K* value was significantly higher than that expected (*P* = 0.019) using a two-tailed test. A higher than expected *K* value indicates there is phylogenetic signal in species-habitat associations. In other words closely related species tended to be more similar in their habitat associations than expected.

### Community phylogenetic structures in different habitat types

**Figure 3:**
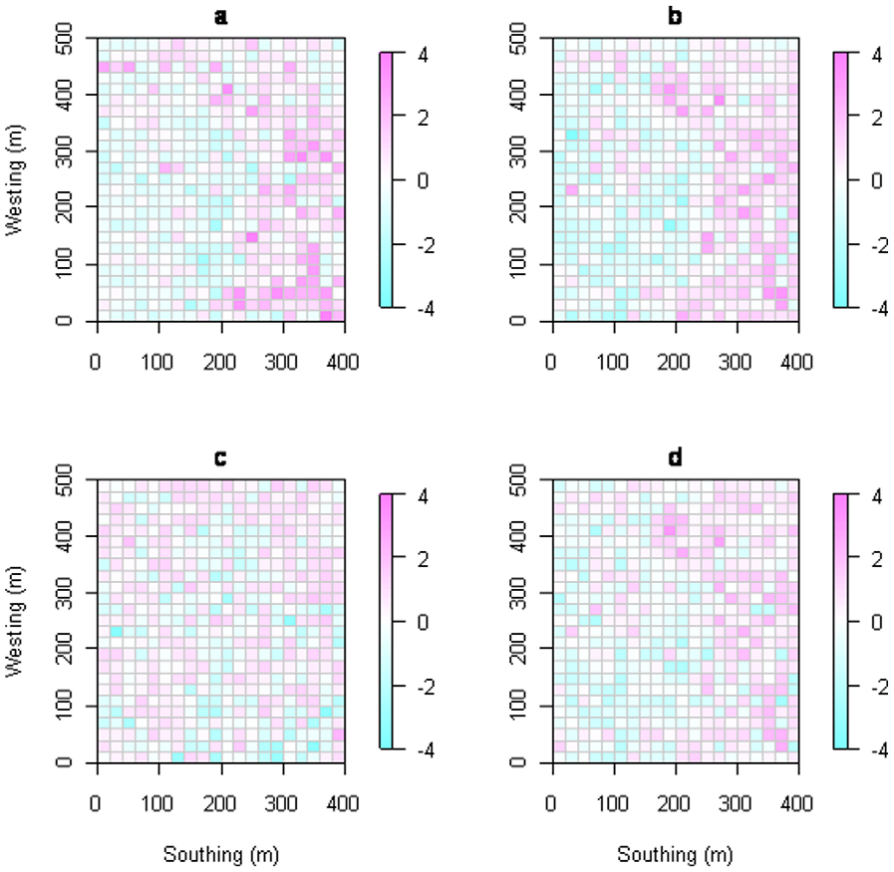
The spatial patterns of NRI and NTI values in the forest plot. Values of NRI and NTI for each 400 m^2^ quadrat in the 20-ha forest dynamics plot in Dinghushan, south China, are calculated using the molecular phylogeny and the Phylomatic phylogeny. Negative NRI and NTI values indicate phylogenetic overdispersion and positive values indicate phylogenetic clustering. The color scales across all NRI and NTI maps are made equivalent to allow for direct visual comparisons between the four maps. a. Barcode NRI; b. Phylomatic NRI; c. Barcode NTI; d. Phylomatic NTI

A total of five habitat types were classified in the DHS FDP and they are mapped using different colors in Fig. 2. The Net Relatedness Index (NRI) value and the Nearest Taxon Index (NTI) value in each quadrat is marked in the 20-ha plot in Fig. 3. The results from the Phylomatic phylogeny found phylogenetic clustering in the valley habitat using both the NRI and the NTI metrics, and in the low-slope habitat using the NRI metric. Phylogenetic overdispersion was found in the high-gully, the high-slope, and the ridge-top habitats using both the NRI and NTI metrics (Table 4 and Fig. 3). When using the molecular phylogeny, we found significant phylogenetic structuring in six of the 10 tests (two metrics per habitat type). Specifically we found phylogenetic clustering in the valley and the low-slope habitats, phylogenetic overdispersion in the high-slope and the ridge-top habitats, and a phylogenetically random pattern in the high-gully habitat using both the NRI and the NTI metrics.

**Table 4:** The estimated mean and standard error of the NRI and the NTI values in the DHS habitat types estimated using first order simultaneous spatial autoregression for the molecular phylogeny (columns labeled “Molecular NRI/NTI”) or for the Phylomatic phylogeny (columns labeled “Phylomatic NRI/NTI”).

Habitat type/Spatial scale	Molecular NRI	Phylomatic NRI	NRI difference	Molecular NTI	Phylomatic NTI	NTI difference
Valley	0.61±0.10^***^	0.53±0.09^***^	0.08±0.09	**0.02±0.09**	0.40±0.08^***^	−0.38±0.13^**^
High-gully	**−0.07⋅0.13**	−0.45±0.13^**^	0.38±0.13^**^	**−0.01±0.12**	−0.41±0.12^**^	0.40±0.18^*^
Low-slope	0.32±0.09^**^	0.28±0.10^**^	0.04±0.09	0.19±0.08^*^	**0.18±0.10**	0.01±0.14
High-slope	−0.45±0.12^***^	−0.48±0.12^***^	0.03±0.11	−0.28±0.11^*^	−0.47±0.09^***^	0.19±0.14
Ridge-top	−0.57±0.09^***^	−0.65±0.11^***^	0.08±0.13	**−0.11±0.12**	−0.41±0.09^***^	0.30±0.17^*^
20 m×20 m	0.141±0.053^**^	0.022±0.052	0.119±0.048^*^	−0.008±0.047	−0.006±0.046	−0.002±0.069
40 m×40 m	0.104±0.103	0.012±0.097	0.092±0.108	0.009±0.088	−0.023±0.090	0.032±0.140
100 m×100 m	0.071±0.247	−0.003±0.137	0.074±0.261	0.157±0.025	0.174±0.145	−0.017±0.255

*Notes:* The *P* values in the “Molecular NRI/NTI” and “Phylomatic NRI/NTI” columns were calculated using a two-tailed t-test to assess whether the mean NRI and NTI values in the habitat types and spatial scales were higher or lower than expected. Negative values indicate that the observed average NRI or NTI was phylogenetically overdispersed. Positive values indicate that the observed average NRI or NTI score was phylogenetically clustered. The columns labeled “NRI or NTI difference” provided the mean of the difference between the molecular phylogeny and Phylomatic NRI and NTI values in each habitat type or spatial scale and were calculated using a two-tailed paired t-test to assess whether the NRI and NTI values in a habitat type or spatial scale calculated from the barcode phylogeny were significantly different than those calculated using the Phylomatic phylogeny. We found all differences among habitats in NRI and NTI were statistically significant according to the spatial GLS tests (*P*<0.01). The asterisk ^***^, ^**^or ^*^ indicate the significance at the level of *P*<0.001, 0.01 or 0.05 respectively.

**Figure 4:**
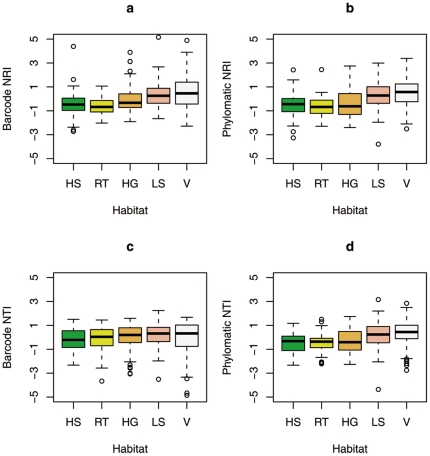
The total distributions of NRI or NTI values in different habitats generated from the molecular and Phylomatic phylogenies. The solid black line across the color box represents the median value. A hollow circle indicates an outlier value of NRI or NTI. HS, High-slope; RT, Ridge-top; HG, High-gully; LS, Low-slope; V, Valley.

When comparing our molecular phylogeny to the less well-resolved Phylomatic tree, five of ten inferences were similar. Analyses based on the molecular phylogeny identified significant phylogenetic structuring in the low-slope habitat using the NTI metric for which the Phylomatic phylogeny did not. In the remaining four cases, the Phylomatic phylogeny demonstrated significant phylogenetic structuring but the molecular phylogeny did not. It is important to note that although the molecular phylogeny generally provided higher NRI and NTI values, this did not necessarily mean these values were more often non-random. For example, an insignificant mean NTI value of −0.01 in the HG habitat was recorded using the molecular phylogeny, but a lower and significant mean NTI value of −0.41 was recorded in this habitat using the Phylomatic phylogeny (Table 4). When directly comparing the NRI and NTI values from the 500 individual quadrats within the five habitat types using paired t-tests, four out of the ten comparisons were significantly different when comparing the results from the molecular and Phylomatic phylogenies (Table 4). We also quantified the NRI and NTI for each habitat type using all species found in a habitat as the assemblage. We found that NRI values from the molecular phylogeny were positive in seven cases and negative in two others cases while NRI values from the Phylomatic phylogeny were positive in two occasions and negative in two cases. The NTI values from the molecular phylogeny were negative in nine cases while the NTI values from the Phylomatic phylogeny were positive in four cases and negative in six cases (Fig. 4).

## Discussion

The niche versus neutral debate is particularly important in tropical forest community ecology given the elevated levels of species richness and often low population sizes of many species in these systems. Previous analyses of tropical tree communities have suggested that lineages non-randomly sort into different habitat types [11] thereby indicating the potential importance of niche-based processes during tropical tree community assembly. Beyond simply finding support for niche-based assembly, this phylogenetically-based analysis was important in that it detected non-random habitat associations that traditional species-based analyses could not detect [8]. This work has been important for our understanding of tropical tree community assembly and for its depiction of the additional information that may be gleaned when using phylogenetic trees. That said, this work comes from a single forest plot in Panama and similar studies from other tropical regions could help determine the generality of these findings. Further, the non-random sorting of lineages into different habitat types in Panama was not integrated with information regarding the degree to which closely related species share similar habitat preferences. In other words whether or not there is phylogenetic signal in species-habitat associations. The present study aimed to quantify whether the phylogenetic structure of a sub-tropical Chinese tree community was non-random across habitats as what has been done in Panama. Next, we quantified whether there was phylogenetic signal in plant-habitat associations – information critical for inferring which ecological process has influenced community assembly the most (Table 1). Finally, previous work has shown that molecular phylogenies generated from three DNA barcode loci may provide substantially different results than those generated using a less-well resolved Phylomatic phylogeny [13]. In this study we compare and contrast the results from a molecular phylogeny to those to a Phylomatic phylogeny to determine whether the results of previous work [13] are generally applicable. In the following we discuss the results of our study with respect to community assembly and the use of molecular versus Phylomatic phylogenies.

### Community Assembly, habitat specialization and species diversity

Phylogenetic investigations of plant communities have been used to determine whether patterns of species co-occurrence have phylogenetic structure [34], [37]. It is recognized that non-random phylogenetic structure (phylogenetic overdispersion or clustering) indeed exists in animal, plant and microbial communities [38], [39], [40] where approximately sixty percent of previous studies have found evidence for phylogenetic clustering in contemporary terrestrial and plant communities [41]. Two general types of niche-based processes can produce these patterns of non-random phylogenetic community structure – environmental filtering and strong negative or positive biotic interactions. Environmental filtering during community assembly dictates that only a small subset of species share similar ecological strategies or niches can co-occur in a given environment. If closely related species have similar strategies or niches, then environmental filtering should produce a pattern of phylogenetic clustering. Conversely, if closely related species have very divergent strategies or niches, then environmental filtering should result in phylogenetic overdispersion. Non-random biotic interactions (i.e. competition, facilitation, etc) dictating community assembly should generate a pattern of phylogenetic overdispersion if closely related species are similar or phylogenetic clustering if closely related species are very dissimilar. Thus while non-random patterns of phylogenetic community structure are indicative of niche-based processes, it is not possible to identify which process without information pertaining to the similarity of closely related species (i.e. phylogenetic signal) (Table 1).

The present study found that many species (>10%) have a significant positive or negative association with a habitat type in the DHS FDP. Subsequent analyses of the phylogenetic signal in species-habitat associations found that there was significant phylogenetic signal. Thus while only some species were significantly associated with a particular habitat, closely related species did on average tend to be associated with similar habitats. Thus any observed patterns of phylogenetic clustering in this system should be indicative of habitat filtering while patterns of phylogenetic overdispersion should be indicative of biotic interactions.

The phylogenetic structuring analyses showed that mean NRI of all 0.04-ha quadrats (i.e. local assemblages) was significantly different from the null expectation of zero (*P* = 0.008). This deviation indicates niche-based community assembly in this forest, but the inferred process varies with the habitat considered. In particular, both the valley and the low-slope habitats had assemblages that were phylogenetically clustered. We therefore infer that habitat filtering drives the assembly of the communities and co-occurrence of species in these two habitats. In the high-slope and ridge-top habitats communities were generally phylogenetically overdispersed indicating a large role for biotic interactions driving the assembly and co-occurrence of species in these habitats. Interestingly these habitats are apt to suffer drought and they likely have low soil nutrients concentrations. Thus it is possible that facilitation influences co-occurrence and therefore generates a pattern of dissimilar co-occurring species. Lastly, the high-gully species assemblages were no different from those expected by chance, which suggests one of two possibilities. First neutrality may dominate the assembly process in these habitats. Second the strength of habitat filtering is ‘balanced’ by the strength of biotic interactions resulting in a random pattern from non-random processes acting in opposing directions [15].

The results showed that the phylogenetic structure of the species in an entire habitat type often mirrored those found in individual quadrats within that habitat type (Table S2). This finding suggests that the filtering of lineages at the ‘habitat-scale’ largely explains the local-scale phylogenetic pattern. We do note, however, that in some instances this was not the case where the habitat-scale pattern was not found locally. This suggests that non-random ecological interactions within habitats may play a large role in determining local phylogenetic structure.

### Comparative analyses of molecular and Phylomatic phylogenies

Previous work has suggested that the lack of terminal resolution in phylogenies generated by the informatics program Phylomatic may bias investigations of community phylogenetic structure [13], [20]. One of these studies was conducted in Panama [13] and the other was simulation based [20]. Therefore it is unclear how generalizable the findings are to other systems. Thus additional studies that compare the results generated from the resolved molecular phylogenies to those from a Phylomatic phylogeny are needed. The present study has performed such a comparison.

The results from the molecular phylogeny generated from three DNA barcode loci had higher values of both NRI and NTI metrics than those generated using the Phylomatic tree in nine out of ten comparisons (Table 4). In other words the results from the resolved molecular phylogeny tended to show more phylogenetic clustering than that found using the Phylomatic phylogeny. This result is similar to that found in Panama [12] and in previous simulation work [20] that suggests that increased resolution provided by molecular phylogenies should allow for the detection of non-random phylogenetic community structure that a Phylomatic phylogeny cannot detect. In other words, the lack of resolution in a Phylomatic phylogeny likely leads to Type II statistical errors.

### Conclusions

The present study sought to determine whether niche-based or neutral processes dominate the assembly of tree communities in a sub-tropical Chinese forest. The work quantified the phylogenetic structure of tree communities in five habitat types and the phylogenetic signal in plant-habitat associations. Using a conceptual framework that integrates the level of phylogenetic signal in plant-habitat associations with the phylogenetic dispersion of species in a community (Table 1) we infer that niche-based processes (habitat filtering and facilitation) drive the assembly of communities in this forest. These results are consistent with findings from a similar study in Panama [13] suggesting that local-scale niche-based processes are important in both of these regions despite their very different biogeographic histories and regional species pool compositions. The work also provides further evidence that less well-resolved Phylomatic phylogenies tend to generate Type II statistical errors and that utilizing resolved molecular phylogenies is therefore advised when feasible. It is suggested that the feasibility of generating such molecular community phylogenies is enhanced through the utilization of sequence data from three commonly used DNA barcoding loci (*rbcL*, *trnH-psbA*, and *matK*). We expect that as barcoding becomes more widespread, community phylogenetics researchers will benefit from ‘tapping into’ the vast resource that is a DNA barcode library.

## Supporting Information

Table S1 **A list of taxa, GenBank accession numbers, and tree tag numbers.**
(DOC)

Table S2 **The estimated mean and standard error (S.E.) of the NRI and NTI values in the DHS habitat types estimated using first order simultaneous spatial autoregression for the barcode phylogeny (columns labeled “Barcode NRI/NTI”) or for the phylomatic phylogeny (columns labeled “Phylomatic NRI/NTI”), using habitat sample files.**
(DOC)

Text S1 **Sequence editing and alignment of three barcode loci.**
(DOC)

Text S2 **A description of which individual species were associated with particular habitat types.**
(DOC)
