# Supplementary material for: Exploring Tree-Habitat Associations in a Chinese Subtropical Forest Plot Using a Molecular Phylogeny Generated from DNA Barcode Loci
Source: PLoS One. 2011 Jun 20;6(6):e21273. doi: 10.1371/journal.pone.0021273 (PMC3119057; doi:10.1371/journal.pone.0021273)
Supplement: Table S1 — A list of taxa, GenBank accession numbers, and tree tag numbers. (DOC) [file pone.0021273.s001.doc]

**Exploring Tree-Habitat Associations in a Chinese Subtropical Forest Plot Using a Molecular Phylogeny Generated from DNA Barcode Loci**

Nancai Pei, Ju-Yu Lian, David L. Erickson, Nathan G. Swenson, W. John Kress, Wan-Hui Ye, Xue-Jun Ge

**Table S1** A list of taxa, GenBank accession numbers, and tree tag numbers.

| **Taxon** | **Family** | **Order** | ***rbcL*** | ***matK*** | ***psbA-trnH*** | **Tree Tag Number** |
| --- | --- | --- | --- | --- | --- | --- |
| *Aralia_spinifolia* | Araliaceae | Apiales | HQ415059 | HQ415247 | HQ415411 | 1916x |
| *Schefflera_octophylla* | Araliaceae | Apiales | HQ415082 |  | HQ415433 | 0822047 |
| *Pittosporum_glabratum 1* | Pittosporaceae | Apiales | HQ415091 | HQ415274 | HQ415442 | 1910124 |
| *Pittosporum_glabratum 2* | Pittosporaceae | Apiales | HQ415079 | HQ415263 | HQ415430 | 0719x |
| *Ilex_chapaensis* | Aquifoliaceae | Aquifoliales | HQ415069 | HQ415254 | HQ415421 | 0909120 |
| *Ilex_cochinchinensis* | Aquifoliaceae | Aquifoliales | HQ415071 | HQ415256 | HQ415423 | 0719039 |
| *Ilex_ficoidea* | Aquifoliaceae | Aquifoliales | HQ415067 | HQ415253 | HQ415419 | 0223x |
| *Ilex_macrocarpa* | Aquifoliaceae | Aquifoliales | HQ415064 |  | HQ415416 | 0118012 |
| *Ilex_memecylifolia* | Aquifoliaceae | Aquifoliales | HQ415065 | HQ415252 | HQ415417 | 0623015 |
| *Ilex_pubescens* | Aquifoliaceae | Aquifoliales | HQ415066 |  | HQ415418 | 0722044 |
| *Ilex_rotunda* | Aquifoliaceae | Aquifoliales | HQ415070 | HQ415255 | HQ415422 | 0824031 |
| *Ilex_triflora* | Aquifoliaceae | Aquifoliales | HQ415068 |  | HQ415420 | 1716021 |
| *Caryota_maxima* | Arecaceae | Arecales | HQ415227 | HQ415391 | HQ415573 | 0321015 |
| *Euonymus_laxiflora* | Celastraceae | Celastrales | HQ415198 | HQ415365 | HQ415546 | 0823101 |
| *Euonymus_nitidus* | Celastraceae | Celastrales | HQ415197 | HQ415364 | HQ415545 | 1518193 |
| *Viburnum_odoratissimum* | Caprifoliaceae | Dipsacales | HQ415114 | HQ415292 | HQ415464 | 0115067 |
| *Diospyros_eriantha* | Ebenaceae | Ericales | HQ415185 | HQ415355 | HQ415534 | 0519014 |
| *Diospyros_morrisiana* | Ebenaceae | Ericales | HQ415184 | HQ415354 | HQ415533 | 0921044 |
| *Craibiodendron_kwangtungense* | Ericaceae | Ericales | HQ415118 | HQ415296 | HQ415468 | 0118002 |
| *Enkianthus_quinqueflorus* | Ericaceae | Ericales | HQ415063 | HQ415251 | HQ415415 | 1924123 |
| *Rhododendron_henryi* | Ericaceae | Ericales | HQ415074 | HQ415258 | HQ415425 | 0823071 |
| *Rhododendron_henryi_*var.*_concavum* | Ericaceae | Ericales | HQ415075 | HQ415259 | HQ415426 | 0623029 |
| *Rhododendron_mariae* | Ericaceae | Ericales | HQ415073 |  |  | 0621027 |
| *Rhododendron_simsii* | Ericaceae | Ericales | HQ415076 | HQ415260 | HQ415427 | 0223x |
| *Maesa_salicifolia* | Maesaceae | Ericales | HQ415072 | HQ415257 | HQ415424 | 0723x |
| *Ardisia_quinquegona* | Myrsinaceae | Ericales | HQ415236 | HQ415400 | HQ415582 | 1417026 |
| *Rapanea_neriifolia* | Myrsinaceae | Ericales | HQ415125 | HQ415303 | HQ415475 | 1616128 |
| *Pentaphylax_euryoides* | Pentaphylacaceae | Ericales | HQ415203 | HQ415369 | HQ415550 | 1009249 |
| *Ternstroemia_gymnanthera* | Pentaphylacaceae | Ericales | HQ415106 | HQ415285 | HQ415456 | 0406135 |
| *Chrysophyllum_lanceolatum_*var.*_stellatocarpon* | Sapotaceae | Ericales | HQ415117 | HQ415295 | HQ415467 | 1616x |
| *Sarcosperma_laurinum_f.* | Sapotaceae | Ericales | HQ415158 | HQ415331 | HQ415507 | 1617012 |
| *Styrax_suberifolius* | Styracaceae | Ericales | HQ415050 | HQ415238 | HQ415402 | 0200020 |
| *Symplocos_adenopus* | Symplocaceae | Ericales | HQ415169 | HQ415340 | HQ415518 | Inside |
| *Symplocos_cochinchinensis* | Symplocaceae | Ericales | HQ415170 | HQ415341 | HQ415519 | 1024054 |
| *Symplocos_lancifolia* | Symplocaceae | Ericales | HQ415167 | HQ415339 | HQ415516 | 1810x |
| *Symplocos_wikstroemifolia* | Symplocaceae | Ericales | HQ415168 |  | HQ415517 | Inside |
| *Cleyera_japonica* | Theaceae | Ericales | HQ415217 | HQ415382 | HQ415563 | 1211087 |
| *Eurya_chinensis* | Theaceae | Ericales | HQ415122 | HQ415300 | HQ415472 | 0619124 |
| *Eurya_macartneyi* | Theaceae | Ericales | HQ415121 | HQ415299 | HQ415471 | 0323122 |
| *Schima_superba* | Theaceae | Ericales | HQ415127 | HQ415305 | HQ415477 | Inside |
| *Adenanthera_pavonina_*var.*_microsperma* | Fabaceae | Fabales | HQ415090 | HQ415273 | HQ415441 | 1023x |
| *Albizia_turgida* | Fabaceae | Fabales | HQ415094 |  | HQ415445 | 0519016 |
| *Archidendron_clypearia* | Fabaceae | Fabales | HQ415100 | HQ415281 | HQ415451 | Inside |
| *Archidendron_lucidum* | Fabaceae | Fabales | HQ415101 | HQ415282 | HQ415452 | 1617071 |
| *Erythrophleum_fordii* | Fabaceae | Fabales | HQ415085 | HQ415268 | HQ415436 | 1916x |
| *Ormosia_fordiana* | Fabaceae | Fabales | HQ415096 | HQ415278 | HQ415447 | 1813x |
| *Ormosia_glaberrima* | Fabaceae | Fabales | HQ415097 | HQ415279 | HQ415448 | 0619029 |
| *Ormosia_semicastrata* | Fabaceae | Fabales | HQ415098 | HQ415280 | HQ415449 | 0921099 |
| *Ormosia_semicastrata_*f.*_pallida* | Fabaceae | Fabales | HQ415095 | HQ415277 | HQ415446 | 1016158 |
| *Xanthophyllum_hainanense* | Polygalaceae | Fabales | HQ415112 | HQ415290 | HQ415462 | 1916081 |
| *Castanopsis_chinensis* | Fagaceae | Fagales | HQ415235 | HQ415399 | HQ415581 | 0723027 |
| *Castanopsis_fissa* | Fagaceae | Fagales | HQ415234 | HQ415398 | HQ415580 | 1718002 |
| *Engelhardia_roxburghiana* | Juglandaceae | Fagales | HQ415111 | HQ415289 | HQ415461 | 0620003 |
| *Aidia_canthioides 1* | Rubiaceae | Gentianales | HQ415144 |  | HQ415494 | 1916129 |
| *Aidia_canthioides 2* | Rubiaceae | Gentianales | HQ415163 |  | HQ415512 | 0824001 |
| *Canthium_dicoccum 1* | Rubiaceae | Gentianales | HQ415225 | HQ415389 | HQ415571 | 0520068 |
| *Canthium_dicoccum 2* | Rubiaceae | Gentianales | HQ415205 | HQ415371 | HQ415552 | 0219010 |
| *Canthium_horridium* | Rubiaceae | Gentianales | HQ415226 | HQ415390 | HQ415572 | 0912065 |
| *Catunaregam_spinosa* | Rubiaceae | Gentianales | HQ415178 | HQ415349 | HQ415527 | Inside |
| *Diplospora_dubia* | Rubiaceae | Gentianales | HQ415086 | HQ415269 | HQ415437 | Inside |
| *Gardenia_jasminoides* | Rubiaceae | Gentianales | HQ415113 | HQ415291 | HQ415463 | 0219060 |
| *Ixora_chinensis* | Rubiaceae | Gentianales | HQ415123 | HQ415301 | HQ415473 | 1916x |
| *Lasianthus_chinensis* | Rubiaceae | Gentianales | HQ415060 | HQ415248 | HQ415412 | 0317x |
| *Lasianthus_curtisii* | Rubiaceae | Gentianales | HQ415061 | HQ415249 | HQ415413 | 1507x |
| *Nauclea_officinalis* | Rubiaceae | Gentianales | HQ415201 |  |  | 1717061 |
| *Pavetta_hongkongensis* | Rubiaceae | Gentianales | HQ415062 | HQ415250 | HQ415414 | 1202097 |
| *Psychotria_asiatica* | Rubiaceae | Gentianales | HQ415119 | HQ415297 | HQ415469 | 1916126 |
| *Tarenna_mollissima 1* | Rubiaceae | Gentianales | HQ415200 | HQ415367 | HQ415548 | 0018x |
| *Tarenna_mollissima 2* | Rubiaceae | Gentianales | HQ415237 | HQ415401 | HQ415583 | 0324102 |
| *Clerodendrum_cyrtophyllum* | Verbenaceae | Lamiales | HQ415229 | HQ415393 | HQ415575 | 1117078 |
| *Clerodendrum_fortunatum* | Verbenaceae | Lamiales | HQ415230 | HQ415394 | HQ415576 | 0623x |
| *Clerodendrum_japonicum* | Verbenaceae | Lamiales | HQ415231 | HQ415395 | HQ415577 | 1913080 |
| *Vitex_quinata* | Verbenaceae | Lamiales | HQ415126 | HQ415304 | HQ415476 | 1713129 |
| *Cinnamomum_camphora* | Lauraceae | Laurales | HQ415228 | HQ415392 | HQ415574 | 0904158 |
| *Cryptocarya_chinensis* | Lauraceae | Laurales | HQ415103 | HQ415283 | HQ415453 | 1617068 |
| *Cryptocarya_concinna* | Lauraceae | Laurales | HQ415104 | HQ415284 | HQ415454 | 1517027 |
| *Lindera_chunii* | Lauraceae | Laurales | HQ415171 | HQ415342 | HQ415520 | 0620033 |
| *Lindera_communis* | Lauraceae | Laurales | HQ415173 | HQ415344 | HQ415522 | 0719063 |
| *Lindera_metcalfiana* | Lauraceae | Laurales | HQ415172 | HQ415343 | HQ415521 | 0512056 |
| *Litsea_cubeba* | Lauraceae | Laurales | HQ415130 | HQ415307 | HQ415480 | 0224021 |
| *Litsea_rotundifolia_*var.*_oblongifolia* | Lauraceae | Laurales | HQ415128 | HQ415306 | HQ415478 | 1717x |
| *Litsea_verticillata* | Lauraceae | Laurales | HQ415129 |  | HQ415479 | 0407151 |
| *Machilus_breviflora* | Lauraceae | Laurales | HQ415159 | HQ415332 | HQ415508 | 0720075 |
| *Machilus_chinensis* | Lauraceae | Laurales | HQ415162 | HQ415335 | HQ415511 | 0124041 |
| *Machilus_kwangtungensis* | Lauraceae | Laurales | HQ415161 | HQ415334 | HQ415510 | 1708042 |
| *Machilus_liangkwangensis* | Lauraceae | Laurales | HQ415164 | HQ415336 | HQ415513 | 0118x |
| *Machilus_phoenicis* | Lauraceae | Laurales | HQ415160 | HQ415333 | HQ415509 | 0723066 |
| *Machilus_velutina* | Lauraceae | Laurales | HQ415165 | HQ415337 | HQ415514 | 1507x |
| *Machilus_velutina_2* | Lauraceae | Laurales | HQ415134 | HQ415310 | HQ415484 | 1307027 |
| *Neolitsea_aurata* | Lauraceae | Laurales | HQ415213 | HQ415378 | HQ415560 | 0310x |
| *Neolitsea_chuii* | Lauraceae | Laurales | HQ415210 | HQ415375 | HQ415557 | 0819104 |
| *Neolitsea_membranaceum* | Lauraceae | Laurales | HQ415211 | HQ415376 | HQ415558 | 0118x |
| *Neolitsea_umbrosa* | Lauraceae | Laurales | HQ415212 | HQ415377 | HQ415559 | 1407x |
| *Magnolia_paenetalauma* | Magnoliaceae | Magnoliales | HQ415131 | HQ415308 | HQ415481 | 1300060 |
| *Michelia_foveolata* | Magnoliaceae | Magnoliales | HQ415092 | HQ415275 | HQ415443 | 1206106 |
| *Michelia_maudiae* | Magnoliaceae | Magnoliales | HQ415093 | HQ415276 | HQ415444 | 1503036 |
| *Calophyllum_membranaceum* | Clusiaceae | Malpighiales | HQ415099 |  | HQ415450 | 0018x |
| *Garcinia_multiflora* | Clusiaceae | Malpighiales | HQ415192 |  | HQ415541 | 1024x |
| *Garcinia_oblongifolia* | Clusiaceae | Malpighiales | HQ415193 |  |  | 0123050 |
| *Alchornea_trewioides* | Euphorbiaceae | Malpighiales | HQ415177 | HQ415348 | HQ415526 | 0223x |
| *Croton_lachnocarpus* | Euphorbiaceae | Malpighiales | HQ415051 | HQ415239 | HQ415403 | 0221x |
| *Macaranga_bracteata* | Euphorbiaceae | Malpighiales | HQ415215 | HQ415380 | HQ415562 | 1904005 |
| *Macaranga_sampsoni* | Euphorbiaceae | Malpighiales | HQ415216 | HQ415381 |  | 1716062 |
| *Mallotus_apelta* | Euphorbiaceae | Malpighiales | HQ415219 | HQ415383 | HQ415565 | 0013x |
| *Mallotus_hoookerianus* | Euphorbiaceae | Malpighiales | HQ415222 | HQ415386 | HQ415568 | 0407x |
| *Mallotus_paniculatus* | Euphorbiaceae | Malpighiales | HQ415220 | HQ415384 | HQ415566 | 1311183 |
| *Mallotus_philippensis* | Euphorbiaceae | Malpighiales | HQ415221 | HQ415385 | HQ415567 | 1107069 |
| *Sapium_discolor* | Euphorbiaceae | Malpighiales | HQ415199 | HQ415366 | HQ415547 | 1916x |
| *Cratoxylon_cochinchinense* | Hypericaceae | Malpighiales | HQ415110 |  | HQ415460 | Inside |
| *Microdesmis_caseraiaefolia* | Pandaceae | Malpighiales | HQ415208 | HQ415373 | HQ415555 | 1407053 |
| *Antidesma_bunius* | Phyllanthaceae | Malpighiales | HQ415206 |  | HQ415553 | 0523015 |
| *Antidesma_fordii* | Phyllanthaceae | Malpighiales | HQ415204 | HQ415370 | HQ415551 | 1913x |
| *Antidesma_venosum* | Phyllanthaceae | Malpighiales | HQ415207 | HQ415372 | HQ415554 | 0223021 |
| *Aporosa_yunnanensis* | Phyllanthaceae | Malpighiales | HQ415224 | HQ415388 | HQ415570 | 1617125 |
| *Bridelia_fordii* | Phyllanthaceae | Malpighiales | HQ415195 | HQ415363 | HQ415543 | 1914x |
| *Flueggea_virosa* | Phyllanthaceae | Malpighiales | HQ415223 | HQ415387 | HQ415569 | 1818009 |
| *Glochidion_eriocarpum* | Phyllanthaceae | Malpighiales | HQ415188 | HQ415358 | HQ415537 | 1908014 |
| *Glochidion_puberum* | Phyllanthaceae | Malpighiales | HQ415189 | HQ415359 | HQ415538 | 1217x |
| *Glochidion_wrightii* | Phyllanthaceae | Malpighiales | HQ415187 | HQ415357 | HQ415536 | 1202209 |
| *Carallia_brachiata* | Rhizophoraceae | Malpighiales | HQ415233 | HQ415397 | HQ415579 | 0822098 |
| *Casearia_glomerata* | Salicaceae | Malpighiales | HQ415115 | HQ415293 | HQ415465 | 1204041 |
| *Casearia_villilimba* | Salicaceae | Malpighiales | HQ415116 | HQ415294 | HQ415466 | 0720028 |
| *Homalium_cochinchinense* | Salicaceae | Malpighiales | HQ415194 | HQ415362 | HQ415542 | 0622x |
| *Microcos_paniculata* | Malvaceae | Malvales | HQ415136 | HQ415312 | HQ415486 | 0901097 |
| *Pterospermum_heterophyllum* | Malvaceae | Malvales | HQ415057 | HQ415245 | HQ415409 | 1402122 |
| *Pterospermum_lanceaefolium* | Malvaceae | Malvales | HQ415058 | HQ415246 | HQ415410 | 1402124 |
| *Reevesia_thyrsoidea* | Malvaceae | Malvales | HQ415190 | HQ415360 | HQ415539 | 0704175 |
| *Sterculia_lanceolata* | Malvaceae | Malvales | HQ415135 | HQ415311 | HQ415485 | 1219003 |
| *Aquilaria_sinensis* | Thymelaeaceae | Malvales | HQ415056 | HQ415244 | HQ415408 | 1916x |
| *Wikstroemia_indica* | Thymelaeaceae | Malvales | HQ415147 | HQ415322 | HQ415497 | 0324x |
| *Wikstroemia_nutans* | Thymelaeaceae | Malvales | HQ415148 |  | HQ415498 | 0721078 |
| *Blastus_cochinchinensis* | Melastomataceae | Myrtales | HQ415053 | HQ415241 | HQ415405 | 1617029 |
| *Melastoma_sanguineum* | Melastomataceae | Myrtales | HQ415218 |  | HQ415564 | 0324x |
| *Memecylon_ligustrifolium* | Melastomataceae | Myrtales | HQ415087 | HQ415270 | HQ415438 | 1617130 |
| *Memecylon_nigrescens* | Melastomataceae | Myrtales | HQ415088 | HQ415271 | HQ415439 | 0823104 |
| *Acmena_acuminatissima* | Myrtaceae | Myrtales | HQ415209 | HQ415374 | HQ415556 | 1024019 |
| *Rhodomyrtus_tomentosa* | Myrtaceae | Myrtales | HQ415191 | HQ415361 | HQ415540 | 0123045 |
| *Syzygium_buxifolium* | Myrtaceae | Myrtales | HQ415138 | HQ415314 | HQ415488 | 1618186 |
| *Syzygium_champioii* | Myrtaceae | Myrtales | HQ415142 | HQ415318 | HQ415492 | 0524013 |
| *Syzygium_hancei* | Myrtaceae | Myrtales | HQ415140 | HQ415316 | HQ415490 | Inside |
| *Syzygium_jambos* | Myrtaceae | Myrtales | HQ415141 | HQ415317 | HQ415491 | Inside |
| *Syzygium_levinei* | Myrtaceae | Myrtales | HQ415137 | HQ415313 | HQ415487 | 1019080 |
| *Syzygium_rehderianum* | Myrtaceae | Myrtales | HQ415139 | HQ415315 | HQ415489 | 1708234 |
| *Elaeocarpus_decipiens* | Elaeocarpaceae | Oxalidales | HQ415077 | HQ415261 | HQ415428 | 0124040 |
| *Elaeocarpus_japonica* | Elaeocarpaceae | Oxalidales | HQ415080 | HQ415264 | HQ415431 | 1617023 |
| *Elaeocarpus_nitentifolius* | Elaeocarpaceae | Oxalidales | HQ415078 | HQ415262 | HQ415429 | 0319031 |
| *Elaeocarpus_sylvestris* | Elaeocarpaceae | Oxalidales | HQ415081 | HQ415265 | HQ415432 | 0020036 |
| *Sloanea_sinensis* | Elaeocarpaceae | Oxalidales | HQ415102 |  |  | 1010029 |
| *Indocalamus_longiauritus* | Poaceae | Poales | HQ415166 | HQ415338 | HQ415515 | 0223013 |
| *Helicia_cochinchinensis* | Proteaceae | Proteales | HQ415176 | HQ415347 | HQ415525 | 1204031 |
| *Helicia_reticulata* | Proteaceae | Proteales | HQ415175 | HQ415346 | HQ415524 | 0014037 |
| *Artocarpus_styracifolius* | Moraceae | Rosales | HQ415055 | HQ415243 | HQ415407 | 1617x |
| *Ficus_esquiroliana* | Moraceae | Rosales | HQ415152 | HQ415326 | HQ415502 | 1117041 |
| *Ficus_fistulosa* | Moraceae | Rosales | HQ415155 | HQ415328 | HQ415504 | 1508x |
| *Ficus_hirta* | Moraceae | Rosales | HQ415157 | HQ415330 | HQ415506 | 0723058 |
| *Ficus_nervosa* | Moraceae | Rosales | HQ415156 | HQ415329 | HQ415505 | 1615x |
| *Ficus_pandurata* | Moraceae | Rosales | HQ415153 | HQ415327 | HQ415503 | 1212192 |
| *Ficus_superba_*var.*_japonica* | Moraceae | Rosales | HQ415150 | HQ415324 | HQ415500 | 0122027 |
| *Ficus_variegata_*var.*_chlorocarpa* | Moraceae | Rosales | HQ415154 |  |  | 0222005 |
| *Ficus_variolosa* | Moraceae | Rosales | HQ415151 | HQ415325 | HQ415501 | 1615151 |
| *Ficus_vasculosa* | Moraceae | Rosales | HQ415149 | HQ415323 | HQ415499 | Inside |
| *Hovenia_acerba* | Rhamnaceae | Rosales | HQ415232 | HQ415396 | HQ415578 | Inside |
| *Laurocerasus_phaeostica* | Rosaceae | Rosales | HQ415089 | HQ415272 | HQ415440 | 1204046 |
| *Photinia_prunifolia* | Rosaceae | Rosales | HQ415183 |  | HQ415532 | 0224085 |
| *Pygeum_topengii* | Rosaceae | Rosales | HQ415196 |  | HQ415544 | 1812x |
| *Raphiolepis_indica* | Rosaceae | Rosales | HQ415182 | HQ415353 | HQ415531 | 0124097 |
| *Gironniera_subaequalis* | Ulmaceae | Rosales | HQ415052 | HQ415240 | HQ415404 | 1616034 |
| *Trema_tomentosa* | Ulmaceae | Rosales | HQ415174 | HQ415345 | HQ415523 | 1916x |
| *Meliosma_fordii* | Sabiaceae | Sabiales | HQ415133 |  | HQ415483 | 1219010 |
| *Meliosma_rigida* | Sabiaceae | Sabiales | HQ415132 | HQ415309 | HQ415482 | 1201107 |
| *Schoepfia_chinensis* | Schoepfiaceae | Santalaes | HQ415145 | HQ415320 | HQ415495 | 1506x |
| *Schoepfia_jasminodora* | Schoepfiaceae | Santalaes | HQ415146 | HQ415321 | HQ415496 | 1306213 |
| *Toxicodendron_sylvestris* | Anacardiaceae | Sapindales | HQ415143 | HQ415319 | HQ415493 | 0224013 |
| *Canarium_album* | Burseraceae | Sapindales | HQ415083 | HQ415266 | HQ415434 | 0721106 |
| *Canarium_tramdenum* | Burseraceae | Sapindales | HQ415084 | HQ415267 | HQ415435 | 1916x |
| *Acronychia_pedunculata* | Rutaceae | Sapindales | HQ415180 | HQ415351 | HQ415529 | 0822100 |
| *Evodia_lepta* | Rutaceae | Sapindales | HQ415202 | HQ415368 | HQ415549 | 0223x |
| *Glycosmis_parviflora* | Rutaceae | Sapindales | HQ415179 | HQ415350 | HQ415528 | 1716017 |
| *Zanthoxylum_avicennae* | Rutaceae | Sapindales | HQ415109 | HQ415288 | HQ415459 | 0407x |
| *Zanthoxylum_myriacanthum_*f. | Rutaceae | Sapindales | HQ415108 | HQ415287 | HQ415458 | 0702026 |
| *Dimocarpus_longan* | Sapindaceae | Sapindales | HQ415124 | HQ415302 | HQ415474 | Inside |
| *Litchi_chinensis* | Sapindaceae | Sapindales | HQ415120 | HQ415298 | HQ415470 | 1916x |
| *Mischocarpus_pentapetalus* | Sapindaceae | Sapindales | HQ415054 | HQ415242 | HQ415406 | 1718001 |
| *Nephelium_chryseum* | Sapindaceae | Sapindales | HQ415181 | HQ415352 | HQ415530 | 1717108 |
| *Daphniphyllum_oldhami* | Daphniphyllaceae | Saxifragales | HQ415107 | HQ415286 | HQ415457 | 0903038 |
| *Eustigma_balansea* | Hamamelidaceae | Saxifragales | HQ415214 | HQ415379 | HQ415561 | 1408094 |
| *Itea_chinensis* | Iteaceae | Saxifragales | HQ415186 | HQ415356 | HQ415535 | 1204041 |
| *Ehretia_longiflora* | Boraginaceae |  | HQ415105 |  | HQ415455 | 1914014 |

*Note:* Word “Inside” meant that the individual was sampled in the plot, but the tree tag number was not present.
