# Supplementary material for: Exploring Tree-Habitat Associations in a Chinese Subtropical Forest Plot Using a Molecular Phylogeny Generated from DNA Barcode Loci
Source: PLoS One. 2011 Jun 20;6(6):e21273. doi: 10.1371/journal.pone.0021273 (PMC3119057; doi:10.1371/journal.pone.0021273)
Supplement: Table S2 — The estimated mean and standard error (S.E.) of the NRI and NTI values in the DHS habitat types estimated using first order simultaneous spatial autoregression for the barcode phylogeny (columns labeled “Barcode NRI/NTI”) or for the phylomatic phylogeny (columns labeled “Phylomatic NRI/NTI”), using habitat sample files. (DOC) [file pone.0021273.s002.doc]

**Exploring Tree-Habitat Associations in a Chinese Subtropical Forest Plot Using a Molecular Phylogeny Generated from DNA Barcode Loci**

Nancai Pei, Ju-Yu Lian, David L. Erickson, Nathan G. Swenson, W. John Kress, Wan-Hui Ye, Xue-Jun Ge

**Table S2** The estimated mean and standard error (S.E.) of the NRI and NTI values in the DHS habitat types estimated using first order simultaneous spatial autoregression for the barcode phylogeny (columns labeled “Barcode NRI/NTI”) or for the phylomatic phylogeny (columns labeled “Phylomatic NRI/NTI”), using habitat sample files.

| Habitat type | N | Barcode NRI | Phylomatic NRI | Barcode NTI | Phylomatic NTI |
| --- | --- | --- | --- | --- | --- |
| Valley | 173 | 2.5194 ± 0.0386 | 0.2193 ± 0.0118 | 1.6070 ± 0.0531 | -0.5919 ± 0.0106 |
| High-gully | 77 | -0.7569 ± 0.0118 | -1.9543 ± 0.0154 | -0.7049 ± 0.0074 | -2.375 ± 0.0280 |
| Low-slope | 115 | 0.3131 ± 0.0131 | -1.5325 ± 0.0136 | 1.2638 ± 0.0265 | -1.3368 ± 0.0108 |
| High-slope | 73 | -0.4646 ± 0.0108 | 1.2733 ± 0.0200 | -1.1668 ± 0.0124 | 1.2924 ± 0.0180 |
| Ridge-top | 62 | -1.3811 ± 0.0206 | 2.6137 ± 0.0194 | 1.177 ± 0.0125 | 2.8609 ± 0.0327 |
