# Supplementary material for: Exploring Tree-Habitat Associations in a Chinese Subtropical Forest Plot Using a Molecular Phylogeny Generated from DNA Barcode Loci
Source: PLoS One. 2011 Jun 20;6(6):e21273. doi: 10.1371/journal.pone.0021273 (PMC3119057; doi:10.1371/journal.pone.0021273)
Supplement: Text S1 — Sequence editing and alignment of three barcode loci. (DOC) [file pone.0021273.s003.doc]

**Exploring Tree-Habitat Associations in a Chinese Subtropical Forest Plot Using a Molecular Phylogeny Generated from DNA Barcode Loci**

Nancai Pei, Ju-Yu Lian, David L. Erickson, Nathan G. Swenson, W. John Kress, Wan-Hui Ye, Xue-Jun Ge

**Text S1** Sequence editing and alignment of three barcode loci.

The *rbcLa* marker was initially aligned via nucleotide sequence in Chromas. All contigs were screened for the presence of stop codons and ambiguities after which the consensus sequence was added to a global alignment that was check manually. The global *rbcLa* alignment was first exported from CLUSTAL X 2.0 [1] as a FASTA file for use in BLAST searches, and then transformed a second export it into nexus file in Se-Al v2.0a 11 Carbon [2] for phylogenetic reconstruction. For *matK*, sequences were screened in Seqman and all contigs were exported individually (i.e., un-aligned) in FASTA file format. We then used MEGA 4 [3] to perform alignment via back-translation. Back-translation first converts nucleotide sequence into amino acid sequence, and CLUSTAL X performs a global alignment of *matK* using amino acid sequence, with the resulting amino acid alignment then back-translated to nucleotide sequence in an aligned FASTA format. All *matK* sequences were aligned simultaneously with each other in this manner. For *trnH-psbA*, contigs were exported as un-aligned FASTA files. For phylogenetic analysis, FASTA files were partitioned taxonomically by family or order as appropriate, following the methods of Kress et al. [4]. Each set of taxonomically structured sequences was then aligned using Se-Al v2.0a 11 Carbon. Twenty separate files were generated in this way. Different sets assembly of aligned *trnH-psbA* sequences into a nexus file was achieved by combining them with the *rbcLa* sequences in a supermatrix format as described in SI Materials and Methods in Kress et al. [4].

1. Larkin MA, Blackshields G, Brown NP, Chenna R, McGettigan PA, et al. (2007) Clustal W and clustal X version 2.0. Bioinformatics 23:2947-2948

2. Rambaut A (1996) Se-Al: sequence alignment editor <http://evolve.zoo.ox.ac.uk/>

3. Tamura K, Dudley J, Nei M, Kumar S (2007) MEGA4: molecular evolutionary genetics analysis (MEGA) software version 4.0. Mol Biol Evol 24:1596-1599

4. Kress WJ, Erickson DL, Jones FA, Swenson NG, Perez R, et al. (2009) Plant DNA barcodes and a community phylogeny of a tropical forest dynamics plot in Panama. Proc Natl Acad Sci USA 106:18621-18626
