# Supplementary material for: Exploring Tree-Habitat Associations in a Chinese Subtropical Forest Plot Using a Molecular Phylogeny Generated from DNA Barcode Loci
Source: PLoS One. 2011 Jun 20;6(6):e21273. doi: 10.1371/journal.pone.0021273 (PMC3119057; doi:10.1371/journal.pone.0021273)
Supplement: Text S2 — A description of which individual species were associated with particular habitat types. (DOC) [file pone.0021273.s004.doc]

**Exploring Tree-Habitat Associations in a Chinese Subtropical Forest Plot Using a Molecular Phylogeny Generated from DNA Barcode Loci**

Nancai Pei, Ju-Yu Lian, David L. Erickson, Nathan G. Swenson, W. John Kress, Wan-Hui Ye, Xue-Jun Ge

**Test S2** A description of which individual species were associated with particular habitat types.

Out of the 188 species in the DHS FDP, 35 species (21 species with only one stem in the plot) were found in just one habitat type, while 19 species were absent only from one habitat type. Specifically, 15 species (*Macaranga bracteata*, *Antidesma fordii*, *Cratoxylon cochinchinense*, *Flueggea virosa*, and *Mallotus philippensis* belonging to the order Malpighiales; *Ficus nervosa, Ficus hirta* and *Hovenia acerba* belonging to the order Rosales; *Syzygium buxifolium* and *Rhodomyrtus tomentosa* belonging to the order Myrtales; *Eurya chinensis*, *Nauclea officinalis*, *Michelia maudiae*, *Pterospermum heterophyllum*, and *Litchi chinensis* belonging to the orders Ericales, Gentianales, Magnoliales, Malvales, and Sapindales respectively) were found only in the valley habitat; five species (*Ixora chinensis*, *Lindera communis*, *Glochidion puberum*, *Michelia foveolata*, and *Ficus superba var. japonica* belonging to the orders Gentianales, Laurales, Malpighiales, Magnoliales, and Rosales respectively) were found only in the high-gully habitat; six species (*Clerodendrum cyrtophyllum*, *Clerodendrum fortunatum*, and *Clerodendrum japonicum* belonging to the order Lamiales; *Garcinia multiflora*, *Schoepfia chinensis*, and *Dimocarpus longan* belonging to the orders Malpighiales, Santalaes, and Sapindales respectively) were found only in the low-slope habitat; eight species (*Ehretia longiflora*, *Viburnum odoratissimum*, *Pentaphylax euryoides*, *Erythrophleum fordii*, *Cinnamomum camphora*, *Sloanea sinensis*, *Zanthoxylum avicennae*, and *Daphniphyllum oldhami* belonging to the Boraginaceae, Dipsacales, Ericales, Fabales, Laurales, Oxalidales, Sapindales, and Saxifragales respectively) were found only in the high-slope; and one species (*Styrax suberifolius* belonging to the order Ericales) was only found in the ridge-top habitat.

Two species (*Pittosporum glabratum* belonging to the order Apiales, and *Litsea rotundifolia var. oblongifolia* belonging to the order Laurales) were absent from only the high-gully habitat. Two species were absent from the low-slope habitat (*Canarium tramdenum* belonging to the order Ericales, and *Catunaregam spinosa* belonging to the order Gentianales). Two species were absent from the high-slope habitat (*Machilus kwangtungensis* belonging to the order Laurales, and *Calophyllum membranaceum* belonging to the order Malpighiales). A total of 13 species (*Antidesma bunius*, *Bridelia fordii*, *Glochidion wrightii*, *Macaranga sampsoni*, *Mallotus apelta*, and *Microdesmis caseraiaefolia* belonging to the order Malpighiales; *Ficus esquiroliana* and *Trema tomentosa* belonging to the order Rosales; *Caryota maxima*, *Rhododendron simsii*, *Albizia turgida*, *Pavetta hongkongensis*, *and Syzygium hancei* belonging to the orders Arecales, Ericales, Fabales, Gentianales, and Myrtales respectively) were absent from ridge-top habitat; but no species were absent from the valley habitat.
